# Supplementary material for: Pyruvate Dehydrogenase Kinase 1 inhibition mediated oxidative phosphorylation enhancement in cartilage promotes osteoarthritis progression
Source: BMC Musculoskelet Disord. 2023 Jul 20;24:597. doi: 10.1186/s12891-023-06585-6 (PMC10357736; doi:10.1186/s12891-023-06585-6)
Supplement: Supplementary file 1 — Additional file 1. [file 12891_2023_6585_MOESM1_ESM.zip › KEGG.Gsea.1653622432464/gsea_report_for_OA_1653622432464.html]

Report for OA 1653622432464 [GSEA]

| GS  follow link to MSigDB | GS DETAILS | SIZE | ES | NES | NOM p-val | FDR q-val | FWER p-val | RANK AT MAX | LEADING EDGE || 1 | KEGG\_OXIDATIVE\_PHOSPHORYLATION | Details ... | 104 | 0.63 | 2.95 | 0.000 | 0.000 | 0.000 | 3164 | tags=67%, list=25%, signal=88% |
| 2 | KEGG\_PARKINSONS\_DISEASE | Details ... | 104 | 0.57 | 2.67 | 0.000 | 0.000 | 0.000 | 3202 | tags=63%, list=25%, signal=82% |
| 3 | KEGG\_MISMATCH\_REPAIR | Details ... | 23 | 0.63 | 2.14 | 0.000 | 0.001 | 0.002 | 1557 | tags=52%, list=12%, signal=59% |
| 4 | KEGG\_HISTIDINE\_METABOLISM | Details ... | 24 | 0.62 | 2.11 | 0.000 | 0.002 | 0.006 | 3064 | tags=54%, list=24%, signal=71% |
| 5 | KEGG\_HUNTINGTONS\_DISEASE | Details ... | 154 | 0.41 | 2.05 | 0.000 | 0.002 | 0.009 | 3202 | tags=49%, list=25%, signal=64% |
| 6 | KEGG\_ALZHEIMERS\_DISEASE | Details ... | 147 | 0.41 | 2.02 | 0.000 | 0.002 | 0.012 | 2781 | tags=43%, list=22%, signal=54% |
| 7 | KEGG\_NUCLEOTIDE\_EXCISION\_REPAIR | Details ... | 43 | 0.49 | 1.95 | 0.000 | 0.004 | 0.027 | 1696 | tags=40%, list=13%, signal=45% |
| 8 | KEGG\_PYRUVATE\_METABOLISM | Details ... | 34 | 0.52 | 1.95 | 0.002 | 0.004 | 0.028 | 2795 | tags=53%, list=22%, signal=67% |
| 9 | KEGG\_DNA\_REPLICATION | Details ... | 34 | 0.52 | 1.93 | 0.000 | 0.005 | 0.038 | 1557 | tags=41%, list=12%, signal=47% |
| 10 | KEGG\_TYROSINE\_METABOLISM | Details ... | 39 | 0.50 | 1.90 | 0.000 | 0.005 | 0.044 | 2264 | tags=38%, list=18%, signal=47% |
| 11 | KEGG\_PEROXISOME | Details ... | 71 | 0.42 | 1.87 | 0.000 | 0.006 | 0.062 | 3176 | tags=48%, list=25%, signal=63% |
| 12 | KEGG\_GLYCOSYLPHOSPHATIDYLINOSITOL\_GPI\_ANCHOR\_BIOSYNTHESIS | Details ... | 19 | 0.58 | 1.85 | 0.003 | 0.008 | 0.080 | 2801 | tags=58%, list=22%, signal=74% |
| 13 | KEGG\_VALINE\_LEUCINE\_AND\_ISOLEUCINE\_DEGRADATION | Details ... | 41 | 0.47 | 1.81 | 0.000 | 0.010 | 0.113 | 3552 | tags=46%, list=28%, signal=64% |
| 14 | KEGG\_LYSOSOME | Details ... | 108 | 0.38 | 1.79 | 0.000 | 0.013 | 0.149 | 3631 | tags=48%, list=28%, signal=66% |
| 15 | KEGG\_ARGININE\_AND\_PROLINE\_METABOLISM | Details ... | 48 | 0.43 | 1.71 | 0.005 | 0.025 | 0.281 | 2795 | tags=54%, list=22%, signal=69% |
| 16 | KEGG\_SELENOAMINO\_ACID\_METABOLISM | Details ... | 22 | 0.52 | 1.70 | 0.009 | 0.024 | 0.286 | 1885 | tags=41%, list=15%, signal=48% |
| 17 | KEGG\_AMINO\_SUGAR\_AND\_NUCLEOTIDE\_SUGAR\_METABOLISM | Details ... | 38 | 0.44 | 1.68 | 0.008 | 0.027 | 0.333 | 1795 | tags=29%, list=14%, signal=34% |
| 18 | KEGG\_PORPHYRIN\_AND\_CHLOROPHYLL\_METABOLISM | Details ... | 28 | 0.47 | 1.67 | 0.011 | 0.028 | 0.357 | 1050 | tags=32%, list=8%, signal=35% |
| 19 | KEGG\_GLYCOSAMINOGLYCAN\_DEGRADATION | Details ... | 20 | 0.52 | 1.64 | 0.010 | 0.032 | 0.407 | 3318 | tags=55%, list=26%, signal=74% |
| 20 | KEGG\_CARDIAC\_MUSCLE\_CONTRACTION | Details ... | 62 | 0.39 | 1.62 | 0.000 | 0.036 | 0.465 | 4221 | tags=55%, list=33%, signal=81% |
| 21 | KEGG\_VIBRIO\_CHOLERAE\_INFECTION | Details ... | 50 | 0.39 | 1.60 | 0.013 | 0.040 | 0.534 | 1486 | tags=30%, list=12%, signal=34% |
| 22 | KEGG\_N\_GLYCAN\_BIOSYNTHESIS | Details ... | 37 | 0.43 | 1.60 | 0.012 | 0.040 | 0.549 | 1565 | tags=32%, list=12%, signal=37% |
| 23 | KEGG\_PROTEASOME | Details ... | 42 | 0.41 | 1.58 | 0.021 | 0.042 | 0.593 | 3977 | tags=55%, list=31%, signal=79% |
| 24 | KEGG\_PHENYLALANINE\_METABOLISM | Details ... | 17 | 0.50 | 1.58 | 0.035 | 0.043 | 0.621 | 2192 | tags=35%, list=17%, signal=42% |
| 25 | KEGG\_PROPANOATE\_METABOLISM | Details ... | 28 | 0.44 | 1.55 | 0.028 | 0.051 | 0.697 | 3362 | tags=43%, list=26%, signal=58% |
| 26 | KEGG\_CITRATE\_CYCLE\_TCA\_CYCLE | Details ... | 30 | 0.43 | 1.54 | 0.025 | 0.052 | 0.722 | 2629 | tags=43%, list=20%, signal=54% |
| 27 | KEGG\_PURINE\_METABOLISM | Details ... | 135 | 0.31 | 1.53 | 0.006 | 0.054 | 0.742 | 1852 | tags=27%, list=14%, signal=32% |
| 28 | KEGG\_PENTOSE\_PHOSPHATE\_PATHWAY | Details ... | 24 | 0.43 | 1.46 | 0.060 | 0.087 | 0.897 | 976 | tags=25%, list=8%, signal=27% |
| 29 | KEGG\_PYRIMIDINE\_METABOLISM | Details ... | 78 | 0.32 | 1.45 | 0.029 | 0.090 | 0.906 | 1852 | tags=27%, list=14%, signal=31% |
| 30 | KEGG\_BASE\_EXCISION\_REPAIR | Details ... | 31 | 0.39 | 1.44 | 0.056 | 0.094 | 0.923 | 1557 | tags=26%, list=12%, signal=29% |
| 31 | KEGG\_TRYPTOPHAN\_METABOLISM | Details ... | 33 | 0.38 | 1.43 | 0.062 | 0.098 | 0.936 | 3209 | tags=45%, list=25%, signal=60% |
| 32 | KEGG\_GLYCOLYSIS\_GLUCONEOGENESIS | Details ... | 55 | 0.34 | 1.40 | 0.039 | 0.114 | 0.957 | 3207 | tags=33%, list=25%, signal=43% |
| 33 | KEGG\_BUTANOATE\_METABOLISM | Details ... | 33 | 0.38 | 1.38 | 0.076 | 0.126 | 0.971 | 2897 | tags=42%, list=22%, signal=55% |
| 34 | KEGG\_GLUTATHIONE\_METABOLISM | Details ... | 39 | 0.36 | 1.37 | 0.075 | 0.129 | 0.978 | 1260 | tags=23%, list=10%, signal=25% |
| 35 | KEGG\_O\_GLYCAN\_BIOSYNTHESIS | Details ... | 19 | 0.39 | 1.24 | 0.163 | 0.273 | 1.000 | 2035 | tags=32%, list=16%, signal=37% |
| 36 | KEGG\_BETA\_ALANINE\_METABOLISM | Details ... | 21 | 0.37 | 1.22 | 0.202 | 0.298 | 1.000 | 2795 | tags=38%, list=22%, signal=49% |
| 37 | KEGG\_RNA\_POLYMERASE | Details ... | 22 | 0.36 | 1.19 | 0.206 | 0.342 | 1.000 | 1760 | tags=36%, list=14%, signal=42% |
| 38 | KEGG\_HOMOLOGOUS\_RECOMBINATION | Details ... | 27 | 0.33 | 1.16 | 0.255 | 0.407 | 1.000 | 2029 | tags=37%, list=16%, signal=44% |
| 39 | KEGG\_FATTY\_ACID\_METABOLISM | Details ... | 39 | 0.29 | 1.14 | 0.250 | 0.430 | 1.000 | 2795 | tags=28%, list=22%, signal=36% |
| 40 | KEGG\_GLYCINE\_SERINE\_AND\_THREONINE\_METABOLISM | Details ... | 27 | 0.33 | 1.13 | 0.270 | 0.445 | 1.000 | 1518 | tags=22%, list=12%, signal=25% |
| 41 | KEGG\_METABOLISM\_OF\_XENOBIOTICS\_BY\_CYTOCHROME\_P450 | Details ... | 51 | 0.27 | 1.11 | 0.253 | 0.469 | 1.000 | 2050 | tags=22%, list=16%, signal=26% |
| 42 | KEGG\_DRUG\_METABOLISM\_CYTOCHROME\_P450 | Details ... | 54 | 0.27 | 1.10 | 0.255 | 0.479 | 1.000 | 1492 | tags=19%, list=12%, signal=21% |
| 43 | KEGG\_GLYCOSAMINOGLYCAN\_BIOSYNTHESIS\_HEPARAN\_SULFATE | Details ... | 21 | 0.33 | 1.09 | 0.336 | 0.490 | 1.000 | 3497 | tags=48%, list=27%, signal=65% |
| 44 | KEGG\_SPHINGOLIPID\_METABOLISM | Details ... | 30 | 0.30 | 1.09 | 0.328 | 0.490 | 1.000 | 3462 | tags=50%, list=27%, signal=68% |
| 45 | KEGG\_SYSTEMIC\_LUPUS\_ERYTHEMATOSUS | Details ... | 101 | 0.23 | 1.09 | 0.271 | 0.487 | 1.000 | 2902 | tags=28%, list=22%, signal=35% |
| 46 | KEGG\_STARCH\_AND\_SUCROSE\_METABOLISM | Details ... | 34 | 0.29 | 1.08 | 0.333 | 0.499 | 1.000 | 4021 | tags=50%, list=31%, signal=72% |
| 47 | KEGG\_DRUG\_METABOLISM\_OTHER\_ENZYMES | Details ... | 37 | 0.28 | 1.07 | 0.341 | 0.503 | 1.000 | 918 | tags=16%, list=7%, signal=17% |
| 48 | KEGG\_INTESTINAL\_IMMUNE\_NETWORK\_FOR\_IGA\_PRODUCTION | Details ... | 41 | 0.27 | 1.06 | 0.328 | 0.519 | 1.000 | 2840 | tags=29%, list=22%, signal=37% |
| 49 | KEGG\_SNARE\_INTERACTIONS\_IN\_VESICULAR\_TRANSPORT | Details ... | 33 | 0.29 | 1.06 | 0.350 | 0.512 | 1.000 | 2901 | tags=30%, list=22%, signal=39% |
| 50 | KEGG\_GLYCOSAMINOGLYCAN\_BIOSYNTHESIS\_CHONDROITIN\_SULFATE | Details ... | 17 | 0.34 | 1.06 | 0.368 | 0.503 | 1.000 | 3180 | tags=41%, list=25%, signal=55% |
| 51 | KEGG\_CYSTEINE\_AND\_METHIONINE\_METABOLISM | Details ... | 32 | 0.29 | 1.04 | 0.402 | 0.543 | 1.000 | 3393 | tags=38%, list=26%, signal=51% |
| 52 | KEGG\_PENTOSE\_AND\_GLUCURONATE\_INTERCONVERSIONS | Details ... | 16 | 0.34 | 1.02 | 0.401 | 0.572 | 1.000 | 3301 | tags=50%, list=26%, signal=67% |
| 53 | KEGG\_ARACHIDONIC\_ACID\_METABOLISM | Details ... | 48 | 0.25 | 1.02 | 0.409 | 0.565 | 1.000 | 936 | tags=15%, list=7%, signal=16% |
| 54 | KEGG\_EPITHELIAL\_CELL\_SIGNALING\_IN\_HELICOBACTER\_PYLORI\_INFECTION | Details ... | 63 | 0.23 | 0.98 | 0.505 | 0.662 | 1.000 | 750 | tags=14%, list=6%, signal=15% |
| 55 | KEGG\_STEROID\_HORMONE\_BIOSYNTHESIS | Details ... | 44 | 0.25 | 0.96 | 0.501 | 0.691 | 1.000 | 3000 | tags=32%, list=23%, signal=41% |
| 56 | KEGG\_PROXIMAL\_TUBULE\_BICARBONATE\_RECLAMATION | Details ... | 22 | 0.28 | 0.94 | 0.524 | 0.741 | 1.000 | 2148 | tags=27%, list=17%, signal=33% |
| 57 | KEGG\_BIOSYNTHESIS\_OF\_UNSATURATED\_FATTY\_ACIDS | Details ... | 20 | 0.29 | 0.94 | 0.546 | 0.735 | 1.000 | 1985 | tags=25%, list=15%, signal=30% |
| 58 | KEGG\_RNA\_DEGRADATION | Details ... | 46 | 0.23 | 0.91 | 0.618 | 0.788 | 1.000 | 3207 | tags=35%, list=25%, signal=46% |
| 59 | KEGG\_ASTHMA | Details ... | 27 | 0.25 | 0.88 | 0.651 | 0.868 | 1.000 | 2840 | tags=33%, list=22%, signal=43% |
| 60 | KEGG\_PROTEIN\_EXPORT | Details ... | 19 | 0.27 | 0.85 | 0.652 | 0.919 | 1.000 | 1272 | tags=16%, list=10%, signal=17% |
| 61 | KEGG\_FRUCTOSE\_AND\_MANNOSE\_METABOLISM | Details ... | 31 | 0.23 | 0.84 | 0.730 | 0.922 | 1.000 | 4432 | tags=52%, list=34%, signal=78% |
| 62 | KEGG\_CELL\_CYCLE | Details ... | 115 | 0.18 | 0.83 | 0.828 | 0.922 | 1.000 | 1557 | tags=17%, list=12%, signal=19% |
| 63 | KEGG\_VASOPRESSIN\_REGULATED\_WATER\_REABSORPTION | Details ... | 41 | 0.21 | 0.83 | 0.744 | 0.917 | 1.000 | 1578 | tags=17%, list=12%, signal=19% |
| 64 | KEGG\_AMINOACYL\_TRNA\_BIOSYNTHESIS | Details ... | 30 | 0.22 | 0.80 | 0.783 | 0.951 | 1.000 | 1913 | tags=23%, list=15%, signal=27% |
| 65 | KEGG\_OOCYTE\_MEIOSIS | Details ... | 95 | 0.16 | 0.76 | 0.948 | 0.992 | 1.000 | 1589 | tags=16%, list=12%, signal=18% |
| 66 | KEGG\_NICOTINATE\_AND\_NICOTINAMIDE\_METABOLISM | Details ... | 17 | 0.23 | 0.71 | 0.872 | 1.000 | 1.000 | 3515 | tags=47%, list=27%, signal=65% |
| 67 | KEGG\_PRIMARY\_IMMUNODEFICIENCY | Details ... | 34 | 0.18 | 0.69 | 0.939 | 1.000 | 1.000 | 2919 | tags=29%, list=23%, signal=38% |
| 68 | KEGG\_DILATED\_CARDIOMYOPATHY | Details ... | 81 | 0.15 | 0.68 | 0.992 | 1.000 | 1.000 | 3570 | tags=36%, list=28%, signal=49% |
| 69 | KEGG\_REGULATION\_OF\_AUTOPHAGY | Details ... | 27 | 0.20 | 0.67 | 0.930 | 1.000 | 1.000 | 1424 | tags=15%, list=11%, signal=17% |
| 70 | KEGG\_GLYCEROLIPID\_METABOLISM | Details ... | 40 | 0.17 | 0.67 | 0.952 | 1.000 | 1.000 | 3373 | tags=35%, list=26%, signal=47% |
| 71 | KEGG\_MATURITY\_ONSET\_DIABETES\_OF\_THE\_YOUNG | Details ... | 22 | 0.20 | 0.65 | 0.933 | 0.997 | 1.000 | 1008 | tags=14%, list=8%, signal=15% |
| 72 | KEGG\_RETINOL\_METABOLISM | Details ... | 45 | 0.15 | 0.59 | 0.995 | 1.000 | 1.000 | 566 | tags=7%, list=4%, signal=7% |
| 73 | KEGG\_STEROID\_BIOSYNTHESIS | Details ... | 17 | 0.17 | 0.54 | 0.978 | 0.995 | 1.000 | 4005 | tags=41%, list=31%, signal=60% |
Table: Gene sets enriched in phenotype **OA (10 samples) **[plain text format]****
